# A model of individual cognitive degradation based on the formalism of quantum potential wells

**DOI:** 10.3389/fnhum.2026.1909815

**Published:** 2026-10-08

**Authors:** Alexander Yurievich Petukhov, Nikita Sergeevich Morozov, Yury Vasilievich Petukhov

**Affiliations:** 1 Cognitive Security Laboratory, Neimark University, Nizhny Novgorod, Russia; 2 Laboratory of Digital Radio Engineering, Neimark University, Nizhny Novgorod, Russia

**Keywords:** cognitive activity, cognitive degradation, information images, potential well, quantum mechanics, representations, schrödinger equation, wave-packet self-focusing

## Abstract

To develop a model of individual cognitive degradation based on the mathematical apparatus of quantum mechanics from the perspective of the Information Images Theory. The foundations of the Information Images Theory are described. The human mind is represented as a one-dimensional potential well with finite walls of different heights and an internal potential barrier modeling the boundary between consciousness and subconsciousness. The model parameters are determined in accordance with the proposed theoretical framework. As a result, a mathematical apparatus for modeling the cognitive activity of the human brain was developed, a state equation for information images was obtained, and numerical simulations of cognitive degradation processes were performed. The influence of the potential barrier size on the activation of information images during the simulation of cognitive ability testing is demonstrated. At high barrier values (corresponding to a well-functioning cognitive system), focusing occurs with minimal wave-packet spreading. As the barrier height decreases, which models the degradation of the cognitive system, a gradual increase in the spreading of the information-image packet is observed. Under these conditions, the individual experiences greater difficulty in activating information images, and the level of concentration decreases. Future research will focus on the experimental validation of the proposed model and its application to predicting the dynamics of cognitive functions, assessing the degree of cognitive degradation, and related tasks.

## 1 Introduction

The natural-scientific aspects of the influence of environmental informational factors on living systems remain relatively understudied (Aleksandrov, 2011). Numerous new electrophysiological methods have been developed, primarily methods for recording evoked and event-related potentials in experimental behavioral settings. These methods have made it possible to closely investigate the physiological mechanisms underlying individual stages of information processing, including sensory analysis, attention mobilization, image formation, retrieval of memory representations, decision-making, and other cognitive processes. The study of the temporal characteristics of electrophysiological responses to stimuli of different types and under various conditions has, for the first time, enabled chronometric assessment, i.e., the estimation of the duration of individual stages of information processing directly at the level of the brain substrate (Aleksandrov, 2011; Chernavsky, 2009). A considerable number of fundamental findings have also been obtained through classical psychological methods aimed at studying and analyzing the behavior of subjects in various social situations.

The problem of describing the processes of information transmission and processing by an individual is fundamental to modern cognitive science. Relatively recently, unique natural-science models of information transmission between individuals (Chernavsky, 2009; Cohen Kadosh et al., 2011; Lee et al., 2005; Griffith and Greitzer, 2007), models of cognitive activity (Vandekerckhove, 2014; Faugeras and Inglis, 2015; Kooi, 2015; Haazebroek et al., 2011; Velichkovsky et al., 2016; Anokhin, 1989; Pan et al., 2016; Nikolaev et al., 2016), and other related approaches have emerged. However, many of these models are either poorly scalable or insufficiently formalized and therefore do not provide a fundamental explanation of information transmission processes and the distortions that arise as a result of interaction with the external communicative environment.

Another important aspect of human cognitive activity is that human thinking is not based on code-like structures (similar to those used in computer systems) but rather on the interaction of numerous images. Although these images are grounded in specific material processes—namely, the electrical and chemical activity of the human brain—their formal description remains difficult for a number of reasons within the framework of conventional mathematical models (Anokhin, 1989).

Therefore, this paper proposes new methods for describing the activity of information images (which, in turn, model the cognitive activity of an individual) using the mathematical apparatus of quantum physics, specifically the theory of potential wells (Physics of the microcosm, 1980).

The mathematical apparatus of quantum theory has already been applied to model cognitive processes and brain functioning more broadly. Examples include:

1. The Penrose–Hameroff model (Orch OR) (Hameroff and Penrose, 1996).
2. The Ricciardi–Umezawa model and the Dissipative Quantum Model of the Brain (DQMB) (Vitiello, 1995).
3. Quantum models of cognition.

These and many other models have demonstrated the applicability of quantum-theoretical formalisms to the study of cognitive phenomena. In particular, quantum models of cognition employ mathematical concepts from quantum theory, such as superposition, interference, and entanglement, not because the brain is assumed to literally function as a quantum computer, but because these formalisms can provide a more adequate description of certain cognitive phenomena, including the contextuality of judgments, question-order effects in decision-making, and deviations from classical probability rules. This approach is conceptually close to the one adopted in the present study.

As a specific process for modeling, the phenomenon of cognitive degradation was selected. Cognitive degradation is understood as the gradual deterioration of cognitive functions of the brain (Petukhov and Polevaya, 2017; Roshchina et al., 2022), including the abilities related to perception, information processing, memory, thinking, attention, speech, and other aspects of cognitive activity. Such deterioration may lead to a reduced quality of life and difficulties in everyday, professional, and social functioning. Its causes may vary and include aging, various diseases, side effects of pharmacological treatments, and other factors. Consequently, the population affected by such processes is extremely broad, and the development of tools capable of predicting and potentially managing these processes represents a highly relevant scientific challenge.

## 2 Information images theory

Briefly, the proposed theory is based on the concept of a universal cognitive unit of information within the human mind (Anokhin, 1989), namely the information image (representation), as well as the space in which it exists, its topology, and its properties. Information images (hereinafter referred to as IIs) may be defined as representations of objects and events within any feature space (Petukhov and Polevaya, 2017).

Accordingly, the Information Images Theory (hereinafter referred to as IIT) is an approach for describing informational interactions between individuals, as well as a number of human cognitive functions.

Within the framework of IIT, the human mind is represented as a vast structured collection of interacting images/representations that are continuously subjected to external influences.

Within the framework of this theory, a physical-phenomenological approach is employed. Accordingly, an informational image involved in the dynamics of cognitive processes is considered as an object possessing physical properties, which is characteristic of descriptions used in quantum physics (e.g., virtual particles, “holes” as unoccupied states in valence bonds, etc.) (Physics of the microcosm, 1980). Consequently, an object endowed with physical properties should be described using an appropriate mathematical framework that has been successfully applied to physical phenomena exhibiting similar dynamics. In addition, the use of partially phenomenological models and approaches makes it possible, on the one hand, to relate certain model parameters to experimentally measurable quantities and, on the other hand, to retain a certain degree of flexibility in model construction.

For example, we introduce the concept of activity, or conditional “energy” E, to characterize the communicative activity of informational images. Images with higher activity are located “higher” and closer to the periphery of an individual's informational-image space and therefore interact more frequently. Conversely, low-activity images, characterized by lower energy and greater inertia, are located closer to the center of this space and relatively rarely engage in active interactions with external stimuli. Importantly, however, these latter images are assumed to be more closely associated with and to play a greater role in subconscious processes (Petukhov and Polevaya, 2017).

This parameter can be directly linked experimentally to the resource expenditure—or energy expenditure in the physicobiological sense—required by the human cognitive system to activate a particular image. For example, when an individual attempts to recall something that is not currently in an active state, the cognitive resources required for its activation are relatively high compared with those required to activate an informational image that has been activated frequently or recently.

Moreover, IIT provides an alternative perspective on a number of characteristic patterns observed in the human mind and allows some of them to be interpreted and explained more accurately.

It is evident that an information image cannot be transmitted unchanged through communication between individuals. In essence, every II is unique because each individual possesses a specific and unique personal experience.

Furthermore, a person cannot directly transfer an image existing within their own information image space to another individual. To accomplish this, various communication apparatuses are employed, formed through the social superstructure of communication, referred to as the communication field (CF). The CF represents an informational commonality of individual experience and the collective unconscious, formed through an individual's existence within society. Communication apparatuses include speech, visual means of information transmission, tactile communication, symbolic communication, and other modalities.

In previous studies, the mathematical model describing interactions between information images was based on diffusion equations, primarily the Langevin equation. This approach made it possible to describe a number of particular cases of cognitive processes; however, it also possessed certain limitations associated with the specific nature of information images. A more detailed description of IIT, its application to real cognitive processes, and its experimental validation can be found in Petukhov and Polevaya (2017).

However, for the further development of this theory, it was decided to employ the mathematical formalism of quantum theory. This choice is motivated by a number of analogies between the interactions and existence of informational images and certain entities described in quantum theory, particularly virtual particles.

A virtual particle (VP) can be understood as a quantum of a relativistic field involved in vacuum fluctuations. From a general quantum-mechanical perspective, VPs may be regarded as particles arising in intermediate states during particle transitions and interactions. A VP has the quantum numbers of a corresponding real elementary particle with mass m; however, it does not satisfy the usual on-shell relationship between energy E and momentum p.

An analogy can be drawn here with an individual, who also generates a “cloud” of informational images that, taken together, represent their mind as well as their accumulated individual and social experience (Petukhov and Polevaya, 2017).

Furthermore, from the perspective of classical physics, a free particle—that is, a particle not acted upon by external forces and therefore either at rest or moving uniformly in a straight line—cannot spontaneously emit or absorb another particle. For example, a free electron cannot independently emit or absorb a photon, since such processes would violate either energy or momentum conservation. An electron at rest has the minimum possible energy and therefore cannot emit a photon, which necessarily carries energy, without violating energy conservation. Similarly, an electron moving at a constant velocity cannot produce a photon solely at the expense of its kinetic energy, because such a process would be incompatible with simultaneous conservation of energy and momentum. The same considerations apply to the absorption of a photon by a free electron.

The situation is described differently within quantum mechanics. According to the uncertainty principle, one of the fundamental principles of quantum theory, for a process occurring over a short time interval Δt, the energy of an intermediate state is not sharply defined. Energy and momentum may therefore exhibit quantum uncertainty, allowing intermediate processes that have no direct classical analog. In heuristic descriptions, this is sometimes expressed in terms of “temporary violations” of classical energy constraints or “local violations” of classical momentum constraints over sufficiently short time and spatial scales (Physics of the microcosm, 1980).

Similarly, it would not be entirely appropriate to apply conservation laws in their conventional physical sense directly to informational images (IIs). The system of informational images as a whole is, of course, constrained by the energetic and material resources of its physical substrate—the individual. However, when individual informational images rather than the entire system are considered, a classical relationship between mass and momentum is clearly not expected to apply.

Interactions between ordinary real particles are, in the framework of quantum field theory, commonly represented through processes involving the exchange of virtual particles. The total energy and momentum of the physical system are conserved between the initial and final states, whereas intermediate virtual states need not satisfy the same on-shell relations as real particles. Thus, an interaction can be represented as the result of various virtual processes occurring over the characteristic timescale of the interaction (Feynman, 1978).

In an analogous manner, interaction between individuals can be represented as a process of transmitting and receiving informational images—that is, their processing and perception. From this perspective, any informational interaction may be described in terms of virtual processes associated with informational images.

Thus, a considerable number of analogies can be identified between particle interactions in quantum field theory and interactions between individuals within the theory of informational images. Such analogies should certainly not be interpreted as evidence of a direct or literal correspondence. Nevertheless, humans are themselves part of the natural world, and it is therefore reasonable to investigate whether general patterns and principles observed in physical systems—not only phenomena associated with virtual particles, but also processes such as diffusion and conservation-related constraints—may have meaningful counterparts in human cognitive activity.

However, whether these analogies have genuine explanatory and predictive value can only be established by applying the corresponding mathematical formalism to generate specific predictions about cognitive processes and subsequently testing these predictions experimentally.

## 3 Potential well

Since we are considering the information image space, it is natural to introduce coordinates within this space. The particles modeling the movement of information images in this space possess momentum and energy, while the space itself contains local minima of particle potential energy. Therefore, the mind of an individual may be represented as a potential well within which information images perform oscillatory motion. In conventional physics, the typical problem involves quantization of a field inside a potential well and determination of energy levels by solving the stationary Schrödinger equation (Roshchina et al., 2022; Feynman, 1978; Bohm, 1990; Wilson et al., 2011). In the present case, however, the particles already exist and are known a priori.

A similar approach to using the mathematical formalism of quantum mechanics to model cognitive processes was proposed by the authors in Fluegemann et al. (2026); Huang et al. (2025). In contrast to the approach proposed in the present study, which focuses on interactions between informational images (IIs) and the assessment of cognitive states, Fluegemann et al. developed their framework to model human decision-making based on perception and the accumulation of sensory evidence. Their approach specifically accounts for the influence of cognitive control in a two-alternative forced-choice task. The authors demonstrated that the quantum model was able to capture the U-shaped relationship between task accuracy and the level of cognitive arousal. These findings suggest that the mathematical formalism of quantum theory is conceptually suitable for modeling cognitive processes and provide indirect support for the theoretical assumptions underlying the approach proposed in the present study.

The simplest representation of the potential well is shown in Figure 1. The potential energy as a function of coordinate (x) is given by Equation (1).

$$\begin{array}{l} U\left(x\right)=\;\{\begin{array}{c} 0,-\;\frac{a}{2}<x<\frac{a}{2}\left(area\;2\right)\\ U_{0},x\geq \frac{a}{2}\;\left(area\;3\right)\\ U_{0},\;\;\;x\leq -\frac{a}{2}\;\left(area\;1\right) \end{array}, \end{array}$$ (1)

Where, *U*_0_ – is the activity/energy level of an information image required to escape the well.

**Figure 1:**
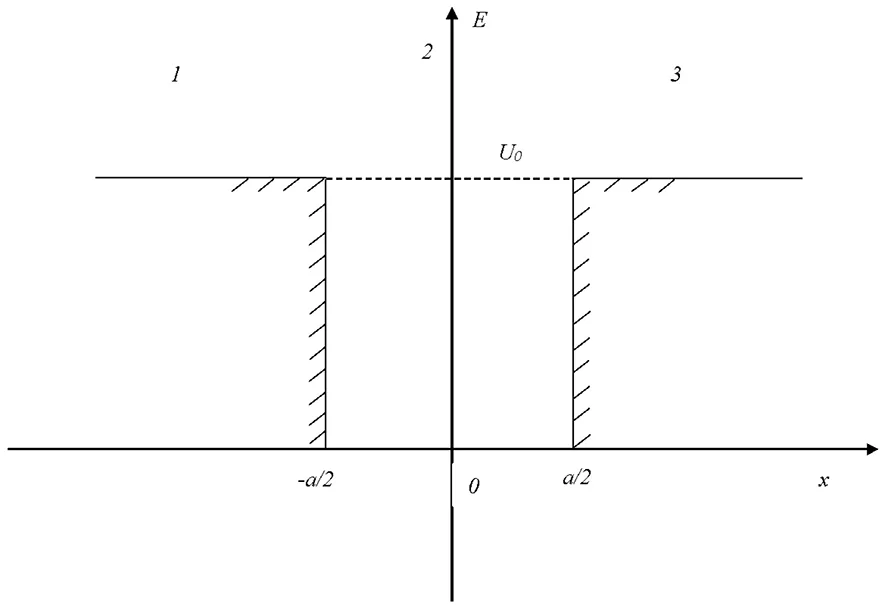
One-dimensional potential well with finite walls. The simplest version.

The overcoming of the potential barrier represented by the walls of the well models informational interaction of the individual, i.e., the excitation of a particle and its transition from one energy level to a higher one. The higher the barrier, the greater the number of energy levels contained within the well and, consequently, the larger the number of information images with different energy values. This corresponds to a better state of the individual's cognitive system and a greater ability to activate larger packets of information images.

The boundaries of the well do not limit the set of information images themselves; rather, they act as a barrier to external communicative interaction. Naturally, this barrier may vary over time depending on the individual's functional state and other factors. However, in the present study we consider very short time intervals and therefore assume the barrier to remain constant during the interaction, such as a brief informational stimulus, a short question, or an image observed on a screen.

A more interesting case is a potential well with asymmetric walls and an additional internal barrier. Such a barrier and wall inhomogeneity model certain specific properties of the human mind, including the conventional division between consciousness and subconsciousness, as well as the complexity of interaction with the external environment.

From the perspective of the Information Images Theory (IIT), consciousness is defined as a structure of information images that interact consciously for the individual, whose informational influence on the external environment results from the deliberate activation of those images. Subconsciousness, in contrast, contains images that do not participate, or participate only minimally, in conscious activity (Petukhov and Polevaya, 2017; Petukhov et al., 2022).

Theoretically, depending on the psychological state of the modeled individual (or a particular cognitive function), such barriers may possess a more complex structure and may occur repeatedly, as illustrated in Figure 2.

$$\begin{array}{ll} U\left(x\right)= & U_{1},\;x\geq \frac{a}{2}\;0,\;\varepsilon <x<\frac{a}{2}\;U_{2},\;-\varepsilon \leq x<\leq \varepsilon \;0,\\ & -\frac{a}{2}<x<-\varepsilon \;\;\;U_{0},\;x\leq -\frac{a}{2}\;,\;\;\;\; \end{array}$$ (2)

Thus, the influence of the external informational environment on the individual's mind may be described as an external action on particles representing information images within a potential well. Preliminary considerations of such a model were presented in earlier studies (Petukhov et al., 2022); however, they were limited to qualitative analysis and did not include numerical calculations, self-oscillatory mathematical formalism, or a rigorous adaptation of the specific parametric framework of IIT (Petukhov and Petukhov, 2022). In the present study, these ideas are further developed through numerical modeling based on the proposed framework.

**Figure 2:**
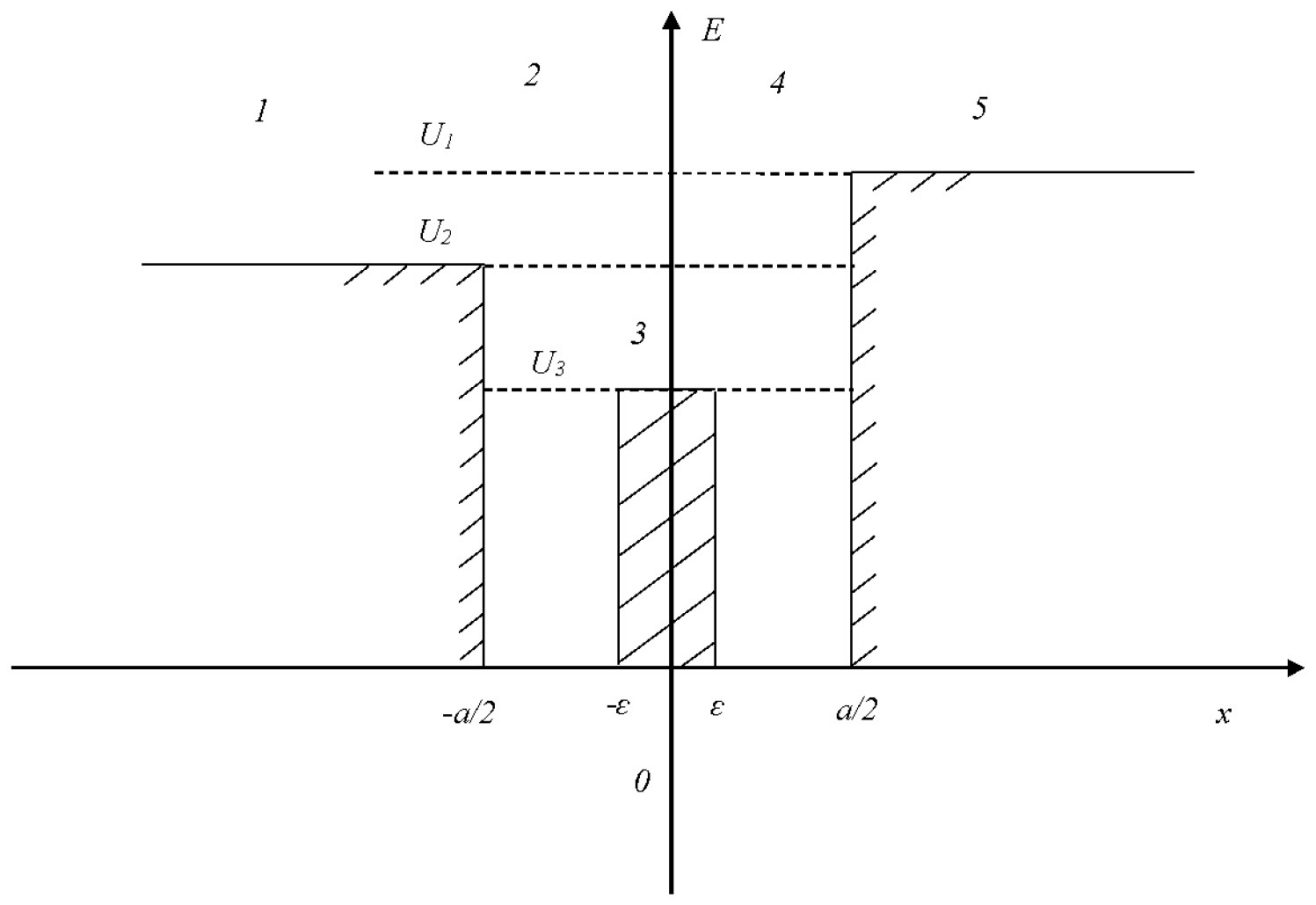
One-dimensional potential well with finite walls. A configuration with walls of different heights and an internal potential barrier.

Let us now write the equations corresponding to the case shown in Figure 1.

The Schrödinger equation outside the potential well for a particle/information image (II) takes the form:

$$\begin{array}{l} \frac{d^{2}\psi_{out}\left(x\right)}{dx^{2}}-\frac{2m}{\hbar^{2}}\left(U_{0}-E\right)\psi_{out}=0,\; \end{array}$$ (3)

Where,

ψ_*out*_− is the wave function outside the potential well.

*E* – is the total energy of the II.

*m* – is the complexity of the II (within the model it corresponds to particle mass; however, the information image is not regarded as a material object).

ℏ – is a conditional constant replacing Planck's constant. Clearly, the value of this constant is not necessarily related to the physical concept of Planck's constant, since the situations and environments considered here are fundamentally different. Nevertheless, within the framework of the proposed model it is reasonable to assume the existence of analogous constants whose values may be determined experimentally.

Introducing the interaction coefficient *k*_1_,

$$\begin{array}{l} k_{1}=\sqrt{\frac{2m}{\hbar^{2}}\left(U_{0}-E\right)}, \end{array}$$ (4)

Using Equation (4), Equation (3) can be rewritten as Equation (5):

$$\begin{array}{l} \frac{d^{2}\psi_{out}\left(x\right)}{dx^{2}}-{k_{1}}^{2}\psi_{out}=0, \end{array}$$ (5)

The solutions of Equation (5) for Region 1 take the form:

$$\begin{array}{ll} \psi_{1}\left(x\right)= & A_{1}e^{k_{1}x}+B_{1}e^{-k_{1}x},\\ \psi_{3}\left(x\right)= & A_{3}e^{k_{1}x}+B_{3}e^{-k_{1}x},\; \end{array}$$ (6)

To ensure that the wave function remains bounded (a necessary condition for realistic information images), the coefficients *B*_1_ and *A*_3_ = 0.

For Region 2, located inside the potential well (i.e., within the human mind), the equation takes the form Equation (5):

$$\begin{array}{l} \frac{d^{2}\psi_{2}\left(x\right)}{dx^{2}}+\frac{2m}{\hbar^{2}}\left(E\right)\psi_{2}=0,\; \end{array}$$ (7)

The interaction coefficient *k*_2_ is given by,

$$\begin{array}{l} k_{2}=\sqrt{\frac{2m}{\hbar^{2}}E}, \end{array}$$

And the corresponding solution of Equation (5) takes the form:

$$\begin{array}{l} \psi_{2}\left(x\right)=Csin\left(k_{2}x+\alpha \right), \end{array}$$ (8)

Thus, the ≪wave≫ functions (6)–(8) describing an information image within the individual's mind are:

$$\begin{array}{ll} \psi_{1}\left(x\right)= & A_{1}e^{k_{1}x},\;\\ \psi_{2}\left(x\right)= & Csin\left(k_{2}x+\alpha \right),\;\\ \psi_{3}\left(x\right)= & B_{3}e^{-k_{1}x}, \end{array}$$ (9)

External influence is represented by a perturbation (U(x)), which is added to the Schrödinger Equation:

$$\begin{array}{l} \frac{d^{2}\psi \left(x\right)}{dx^{2}}-\frac{2m}{\hbar^{2}}\left(U\left(x\right)+V\left(x\right)-E\right)\psi =0,\;\; \end{array}$$ (10)

The perturbation may be specified in different ways depending on its nature, for example by a normal distribution. In most cases this is justified because the combined effect of random influences leads to a Gaussian distribution, as follows directly from the central limit theorem (Petrov, 1976).

It should be noted that most of the parameters introduced for information images are treated as constants. Only the activity/energy level changes during interaction. This assumption is not entirely adequate for a cognitive system that learns, processes information, modifies existing information images, and generates new ones. Nevertheless, the present formulation corresponds to a very short interaction interval and therefore significantly simplifies the problem. Future developments of the model will attempt to address this limitation by introducing additional functions describing changes in information-image parameters resulting from external influences and interactions with other information images.

## 4 Mathematical model

As a physical analogy for the process under consideration, the behavior of wave packets of highly excited atomic states was selected (Fluegemann et al., 2026; Huang et al., 2025). Such systems exhibit interesting effects of successive focusing and defocusing of the wave packet (depending on the model parameters), which may provide a close representation of the processes of activation and organization of information images (focusing) during the execution of a cognitive task performed repeatedly over time. In other words, this situation may be interpreted as a subject repeatedly taking the same well-known test (i.e., without a learning effect) at significant time intervals, for example once every several months, while the success of task completion is measured.

For a healthy individual not undergoing progressive cognitive degradation, the test performance should remain approximately unchanged (Roshchina et al., 2022). If the intervals between testing sessions become substantially longer, performance may gradually decrease due to loss of proficiency. However, if the individual experiences progressive cognitive degradation (caused by disease, medication effects, age-related changes, etc.), negative changes become noticeable even when the intervals between test sessions are relatively short (Figure 3, Schaie et al., 2004; Taylor et al., 2007).

**Figure 3:**
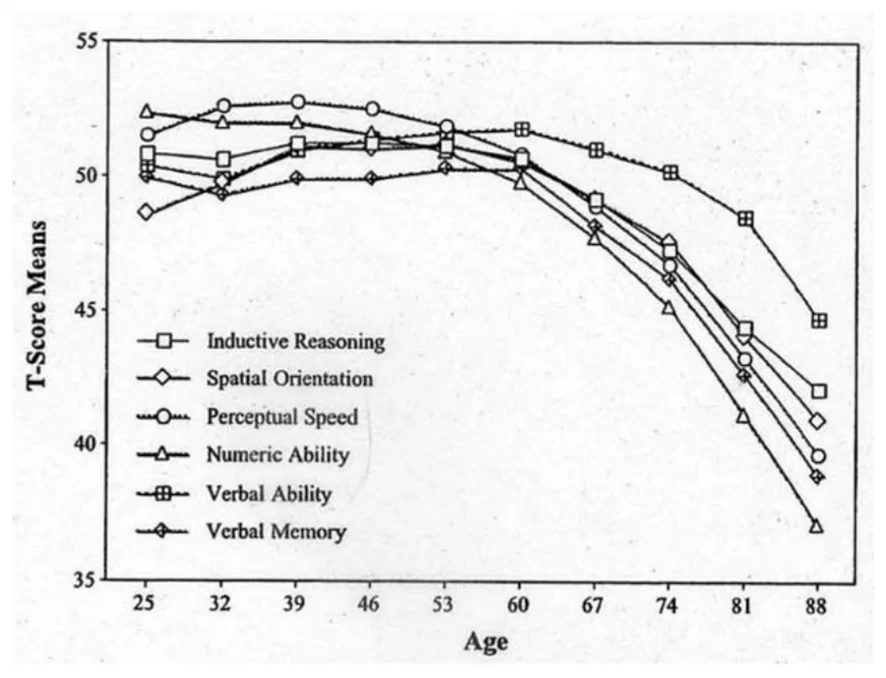
Age-related changes in different cognitive functions. Figure 3 is reproduced from Schaie et al. (2004).

Obviously, the decline in cognitive abilities is a continuous process. Within the framework of the present model, however, it is measured discretely, since the model is intended to represent a testing procedure, i.e., the activation of a group of information images required for solving a particular task (focusing in physical terms). This assumption will be examined using the proposed mathematical model.

Accordingly, the problem is formulated as the study of focusing and defocusing of a group (packet) of activated information images (modeled as a bundle of harmonic oscillations) (Averbukh et al., 1991) located inside a potential barrier, which in turn represents the cognitive space of an individual undergoing testing.

The defocusing function *J* of such a group, depending on the normalized position within the cognitive space *z*ε *(0*, π*)* and time τ, is described by Equation (11):

$$\begin{array}{l} J\left(z,\tau \right)=|\frac{l}{2}\psi \left(z,\tau \right){|}^{2},\;z=x\frac{\pi }{l}, \end{array}$$ (11)

The quantity *l* describes the geometric characteristics of the potential barrier, i.e., the cognitive potential of the individual. Let *N* denote the number of information images in the group (harmonics within the bundle). The behavior of the information-image packet is described by the wave function ψ(*z*, τ). It is represented as a sum of oscillations of *N* “particles” (information images), where each (n)-th particle (*n*∈[1, *N*]) is an oscillator characterized by a harmonic amplitude envelope *a*_*n*_ and represented as Equation (12):

$$\begin{array}{l} \psi \left(z,\tau \right)=\frac{2}{l}\overset{N}{\underset{n=1}{\sum }}a_{n}sin\left(K_{n}z+\delta \right)exp^{-iK_{n}^{2}\tau },\;\;\;\;\;0\leq \tau <\infty , \end{array}$$ (12)

We begin by studying the behavior of the envelope, namely the function *K*_*n*_, which is determined by the wave Equation (13):

$$\begin{array}{l} K_{n}+\frac{2}{\pi }arcsin\left(K_{n}/\underline{\varkappa_{0}}\right)-n=0,\; \end{array}$$ (13)

The variable ϰ_0_ in this equation is a constant and is expressed as follows:

$$\begin{array}{l} \underline{\varkappa_{0}}=\frac{e\sqrt{2{mU}_{0}}}{\pi \hbar } \end{array}$$

The constant ℏ was introduced earlier as a conditional analog of Planck's constant. The base of the natural logarithm *e* and π are also constants. Consequently, the parameter is determined only by the characteristics of the potential barrier *U*_0_. For further modeling, this function will be considered in the generalized form

$$\begin{array}{l} \underline{\varkappa }=n_{p}\sqrt{U_{0}} \end{array}$$

Where, *n*_*p*_ is a constant proportionality coefficient serving as a scaling parameter in the simulations and does not affect the qualitative behavior of the function.

The first task is to determine the value of the wave parameter *K*_*n*_ for a given value of *N*. Instead of seeking an analytical solution, a numerical solution based on the bisection method was employed, since this method guarantees convergence within a bounded interval. In the generalized form, the equation may be written as *f*(*K*_*n*_) = 0. The function is monotonically increasing (derivative > 0). From the restriction imposed on the argument of the arcsin, it follows that *K*_*n*_ ≤ ϰ.

For large values of ϰ, arcsin(*K*_*n*_/ϰ)≈*K*_*n*_/ϰ, therefore the equation approaches *K*_*n*_+(2/π). Analysis of the equation shows that on the interval [0, n] a root exists and lies between 0 and n. Thus, for all n, the solution may be searched for within the interval [0, n] (provided that *n* ≤ ϰ, otherwise arcsin is undefined). Binary search was used to obtain the numerical solution because of its simplicity and stability. The number of iterations was set to 100, which corresponds to double-precision accuracy. Thus, for any given (large) N and height of the potential barrier U0, the values of the wave functions necessary for further calculations have been numerically determined.

The second term determining the parameters of the harmonic envelope is used to estimate variations in oscillation rate and is given by

$$\begin{array}{l} \delta =arcsin\left(K_{n}/\underline{\varkappa_{0}}\right) \end{array}$$

Once the numerical solution for the previous term has been obtained, the calculation of this quantity becomes straightforward.

## 5 Main equation

Having determined the wave function parameter *K*_*n*_, we can obtain the solution of Equation (14):

$$\begin{array}{l} a_{n}=\frac{\frac{2}{l}\left(2\varepsilon_{1}-\varepsilon_{2}\sin\left(\delta \right)\right)-K_{n}\varepsilon_{2}\cos\left(\delta \right)}{\alpha_{n}^{2}\left[{\left(\frac{2}{L}\right)}^{2}+K_{n}^{2}\right]}, \end{array}$$ (14)

The envelope amplitude parameter *a*_*n*_ is determined by several quantities, each of which represents a separate oscillatory phenomenon Equation (15):

$$\begin{array}{ll} \alpha_{n}= & {\left[1+\frac{2}{\pi \sqrt{{\underline{\varkappa }}^{2}-K_{n}^{2}}}\right]}^{1/2},\;\varepsilon_{1}=sin\left(K_{n}z_{0}+\delta \right),\\ \varepsilon_{2}= & {\left(-1\right)}^{n}exp\left(\frac{2}{L}\left(z_{0}-\pi \right)\right)-exp\left(-\frac{2}{L}z_{0}\right). \end{array}$$ (15)

The parameters required for the final calculation are obtained from the previously derived relations:

$$\begin{array}{l} z_{0}=x_{0}\frac{\pi }{l}\;\\ L=\frac{l}{\pi } \end{array}$$

As in the case of ϰ, these parameters possess a physical interpretation. However, within the cognitive-process model they effectively serve only as scaling coefficients and do not directly affect the qualitative behavior of the information-image packet defocusing function.

Thus, the final calculations were performed using the formula (16):

$$\begin{array}{l} J\left(z,\tau \right)=|\overset{N}{\underset{n=1}{\sum }}a_{n}sin\left(K_{n}z+\delta \right)exp^{-iK_{n}^{2}\tau }{|}^{2}, \end{array}$$ (16)

The independent modeling variables are N, z, τ, and U0; the others are either dependent and are calculated during the modeling process using the formulas specified above, or they are constant parameters.

For convenience in constructing graphical representations, the model parameters were selected such that the scaling coefficients became equal to unity. This normalization makes it possible to obtain the characteristic behavior of the system and identify its fundamental regularities in normalized variables, thereby revealing the intrinsic properties of the observed dependencies without reference to the physical dimensions of the original mathematical framework. The specific values of the parameters and the variable ranges of the independent variables used for the modeling are as follows:

$$\begin{array}{l} N=\left(512,2048,8192\right),U0=\left(1000,10000,\infty \right),z\in \left[0,2\right],\;\tau \in \left[0,0.5\right] \end{array}$$

## 6 Results of the modeling and discussion

For different sets of initial parameters, primarily the size of the potential barrier, numerical simulations were performed.

First, we considered the case of very high barrier walls, which corresponds to a well-functioning cognitive system with a very low level of cognitive degradation (Figure 4).

**Figure 4:**
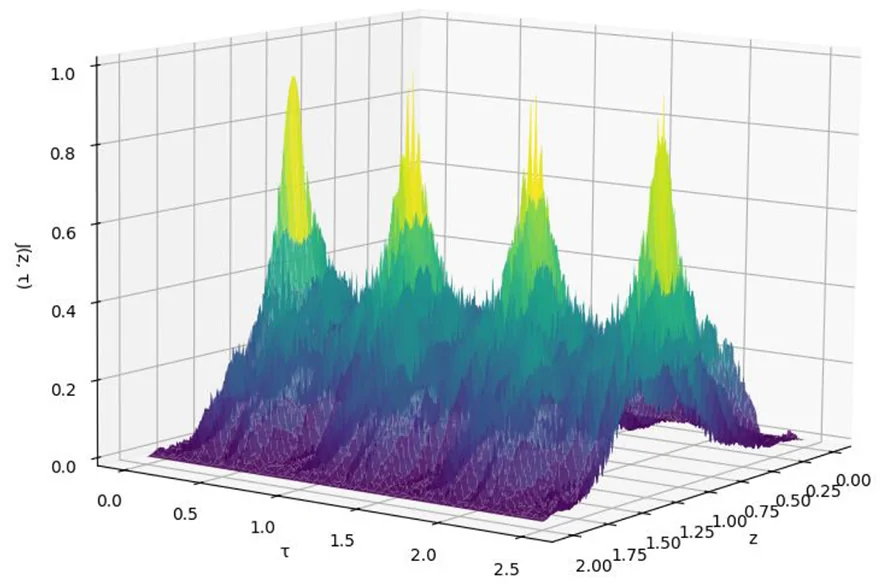
Focusing of a group of information images at relatively high barrier values.

In this case, stable activation (self-focusing) of the information-image packet is observed, while packet spreading remains minimal.

In terms of the proposed interpretation, the individual performs cognitive-function testing with only negligible deterioration in performance. This situation may correspond to a healthy person who is only beginning to experience age-related changes. Under such conditions, the test results are likely to be influenced more strongly by the individual's current state, including fatigue, emotional condition, and similar factors.

Figures 5, 6 present the results obtained for relatively low barrier values. In this case, the spreading of the information-image packet becomes significantly more pronounced, indicating that the individual experiences greater difficulty in performing cognitive-function tests. The overall concentration of packet energy decreases, implying that fewer information images reach the energy levels required for informational interaction. In terms of cognitive testing, this would correspond to an increase in decision-making time and a higher number of errors, since an insufficient number of information images become activated. At the same time, the individual may possess the required information images, but because they remain in a non-activated state, they cannot be effectively utilized. This phenomenon is analogous to situations in which a person knows that certain information has been learned but is temporarily unable to recall it.

**Figure 5:**
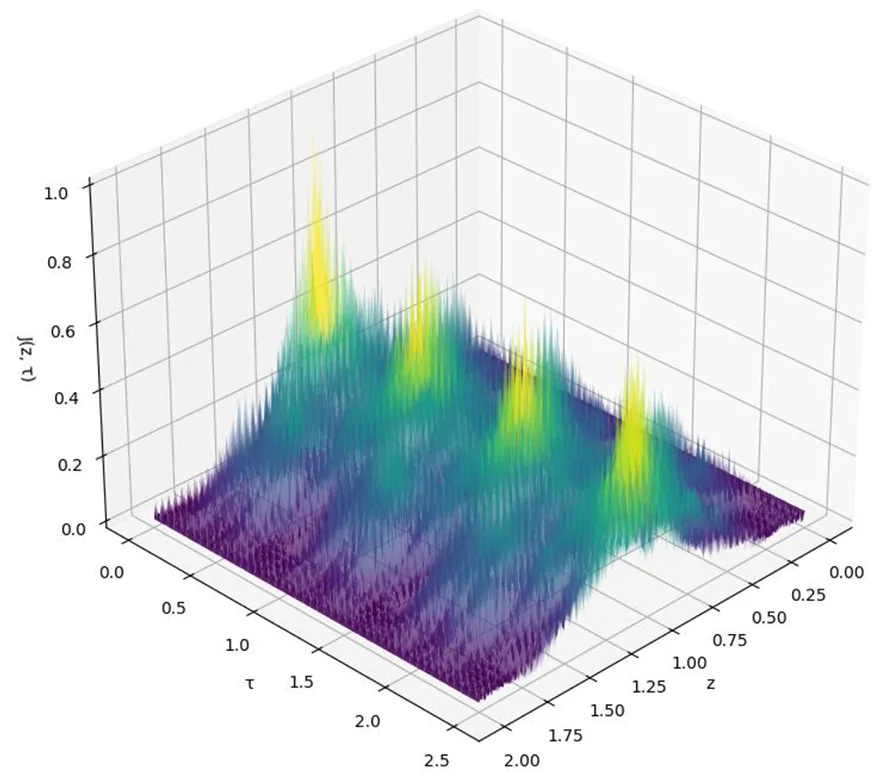
Focusing a group of informational images at a relatively low barrier height. Two result views with the same parameters.

**Figure 6:**
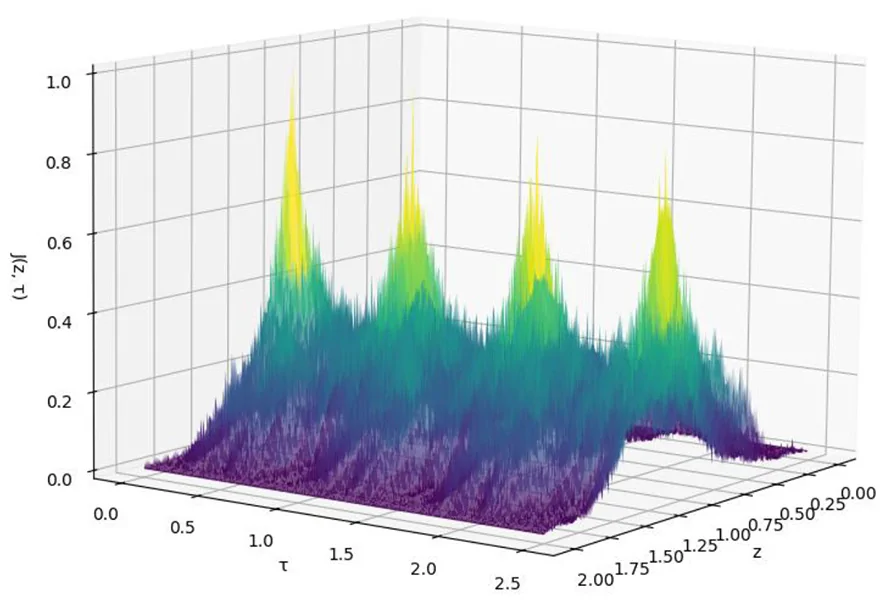
Focusing a group of informational images at a relatively low barrier height. Two result views with the same parameters.

Such activation patterns correspond to individuals experiencing a progressive phase of cognitive degradation, both reversible and irreversible.

Other potential-barrier configurations are also possible, including intermediate cases between the two extremes discussed above. Such examples are presented in Figures 7, 8, where the degree of focusing decreases, but not as rapidly as in Figures 5, 6. A lower barrier than shown in Figures 5, 6 corresponds to a limited cognitive space with significant degradation. If we consider intermediate options between full focusing of the wave packet and progressive defocusing, we can observe the effect of a slight reduction in the peak value of cognitive capabilities without defocusing of the II. Such situations are typical when there is a general decline in the body's tone, and they are not associated with direct degradation of cognitive functions. However, they are also of interest for studying various states of the human cognitive system. Thus, the presented model, even though it is limited to adjusting just one parameter, allows for studying the behavior of a complex cognitive system.

**Figure 7:**
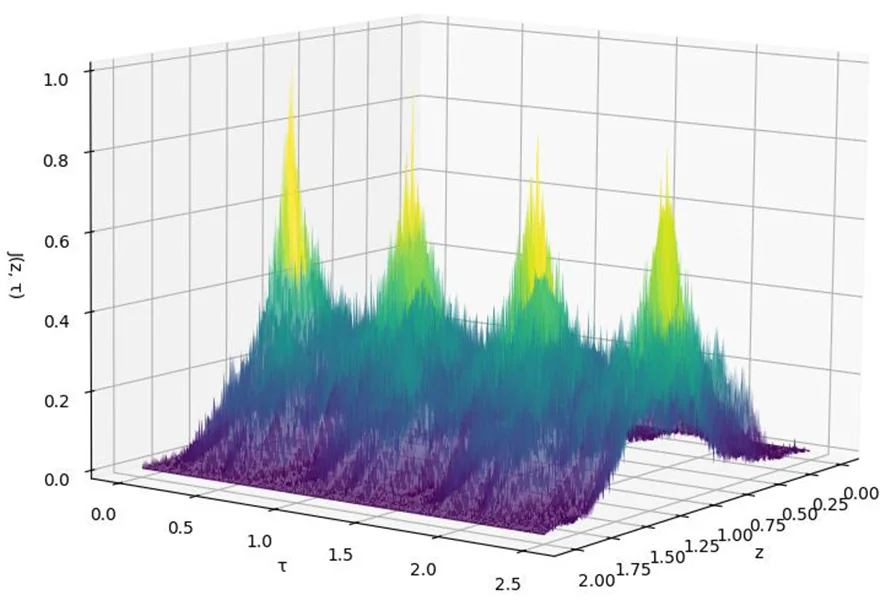
Focusing a group of information images for intermediate relative barrier sizes. Two result views with the same parameters.

**Figure 8:**
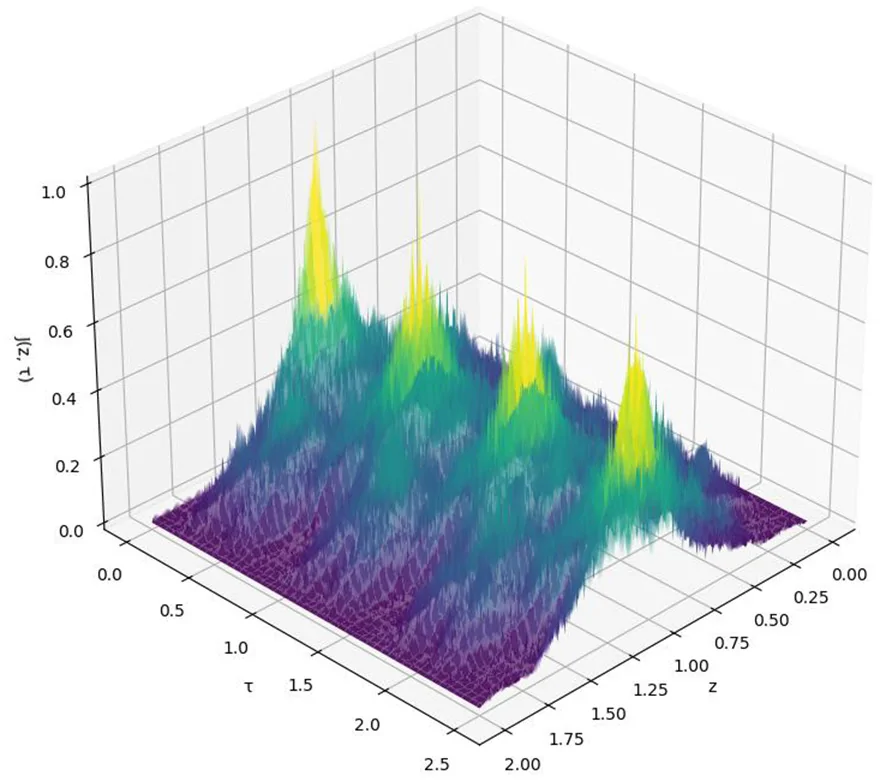
Focusing a group of information images for intermediate relative barrier sizes. Two result views with the same parameters.

Consequently, by varying the size of the potential barrier, it becomes possible to model different states of the human cognitive system. In our model, the size of the potential barrier is directly related to the capabilities of the human cognitive system, because the larger the potential barrier, the more energy levels there are for the II, and consequently, the more informational images, the more different options for being at different levels, and the greater the diversity.

The height of the barrier in an experimental study can be varied in several ways:

1. By comparing groups of individuals with different levels of cognitive ability, as determined by preliminary cognitive testing.
2. By manipulating the participants' state during the experiment—for example, by introducing distractions or inducing stress. Such manipulations would alter the state of the cognitive system and, consequently, the height of the barrier.

Accordingly, the state of the cognitive system would directly affect performance on experimental tasks and, therefore, the measured outcomes. For example, if the Stroop task, a classic cognitive-control and response-inhibition task, were used in the experiment, a decrease in the barrier height would be expected to result in longer response times and a higher error rate (Cohen Kadosh et al., 2011).

Classical models face substantial difficulties in describing cognitive systems in several respects:

1. When information processes are described without treating information objects as physical entities—for example, when they are represented as abstract codes—such models do not account for the specific properties of information as it interacts with the cognitive system, the resulting changes in the system's physical properties, or the fact that information objects are directly related to physical processes through their material substrate, such as electrical impulses and physical media.
2. If information objects are regarded as physical entities, it becomes necessary to account for their specific properties, including those associated with apparent formal violations of conservation laws. These properties are not adequately described by classical, non-quantum mathematical models.
3. Many classical models, including graph-based and probabilistic models, also encounter difficulties in describing the self-organizing states of cognitive systems.

Furthermore, the model presented in this study provides an explanation for such phenomena as the preservation of cognitive performance and memory clarity in individuals who remain professionally active for many years, including after retirement or following injuries. In this interpretation, persistent external factors maintain a high potential barrier, thereby delaying the onset of cognitive degradation. This effect may be particularly pronounced among university professors and educators and is difficult to explain using, for example, the inhibitory-deficit theory or graph-based models.

At the same time, the generalized representation of the potential barrier used in the present study, in which several factors are integrated into a single coefficient, complicates the experimental testing and further refinement of the model. As noted above, future work will involve extending the model by separating the identified factors that influence the qualitative form of the focusing function. Until then, we can only discuss the general consistency of the model's predictions with phenomena observed experimentally.

In the future, it might be possible to develop a function that modifies the potential barrier based on additional conditions (e.g., a person's current state, how well they've slept, their health, their emotional stability, etc.) or even simulates recovery processes with appropriate therapy. Furthermore, the test itself, as well as the conditions under which it's administered, and its specific features, such as the presence of multiple bivalent informational influences (Petukhov et al., 2025), are also important.

## 7 Conclusion

In the course of this study, an original mathematical model of individual cognitive degradation based on the formalism of quantum potential wells and the Information Images Theory was developed. The proposed approach makes it possible to describe the dynamics of information images within the human cognitive space while accounting for their interaction with the external environment.

The influence of the potential-barrier size on the activation of information images during the simulation of cognitive ability testing was demonstrated. At high barrier values, corresponding to a well-functioning cognitive system, focusing occurs with minimal packet spreading. As the barrier height decreases, a gradual increase in the spreading of the information-image packet is observed. Under these conditions, information images become more difficult to activate, and the overall level of concentration decreases.

The scientific novelty of the study also lies in the application of a quantum-mechanical approach to the modeling of cognitive processes, which opens new perspectives for investigating the mechanisms of information processing in the human mind.

The proposed model may be useful for describing both normal and pathological states of the cognitive system. Further development of the model will include the incorporation of additional factors, such as emotional state, stress level, and other variables affecting cognitive activity.

Future work will focus on the experimental validation of the model using real cognitive processes under controlled experimental conditions. The further development of the proposed approach may contribute to the creation of more advanced tools for assessing, predicting, and managing human cognitive functions.

To validate the proposed model and empirically verify it, a comprehensive study is planned, using the following methods:

- Neuropsychological testing with standardized methods for assessing cognitive functions (memory, attention, thinking, executive functions). Special attention will be paid to measuring parameters that correlate with the height of the potential barrier in the model (information processing speed, task accuracy, resistance to interference).
- Longitudinal observation of groups of subjects with different levels of cognitive functioning:
  - healthy subjects of young and elderly age
  - patients with initial signs of cognitive decline
  - individuals with diagnosed neurodegenerative processes
- Cognitive assessments over time using:
  1. working memory tests
  2. tasks assessing cognitive flexibility
  3. methods for evaluating attention switching speed
  4. tests for verbal and non-verbal thinking

Special attention will be paid to studying the possibility of using the model for early diagnosis of cognitive impairments and predicting their progression, which could significantly enhance the effectiveness of preventive and corrective measures.

## Data Availability

The code used to produce the numerical simulations is not publicly available at present but can be made available by the corresponding author upon reasonable request.
